# Protein Structure Prediction: Challenges, Advances, and the Shift of Research Paradigms

**DOI:** 10.1016/j.gpb.2022.11.014

**Published:** 2023-03-30

**Authors:** Bin Huang, Lupeng Kong, Chao Wang, Fusong Ju, Qi Zhang, Jianwei Zhu, Tiansu Gong, Haicang Zhang, Chungong Yu, Wei-Mou Zheng, Dongbo Bu

**Affiliations:** 1 Key Laboratory of Intelligent Information Processing, Institute of Computing Technology, Chinese Academy of Sciences, Beijing 100190, China; 2 University of Chinese Academy of Sciences, Beijing 100049, China; 3 Changping Laboratory, Beijing 102206, China; 4 Microsoft Research AI4Science, Beijing 100080, China; 5 Huawei Noah’s Ark Lab, Wuhan 430206, China; 6 Zhongke Big Data Academy, Zhengzhou 450046, China; 7 Institute of Theoretical Physics, Chinese Academy of Sciences, Beijing 100190, China

**Keywords:** Protein folding, Protein structure prediction, Deep learning, Transformer, Language model

## Abstract

**Protein structure prediction** is an interdisciplinary research topic that has attracted researchers from multiple fields, including biochemistry, medicine, physics, mathematics, and computer science. These researchers adopt various research paradigms to attack the same structure prediction problem: biochemists and physicists attempt to reveal the principles governing **protein folding**; mathematicians, especially statisticians, usually start from assuming a probability distribution of protein structures given a target sequence and then find the most likely structure, while computer scientists formulate protein structure prediction as an optimization problem — finding the structural conformation with the lowest energy or minimizing the difference between predicted structure and native structure. These research paradigms fall into the two statistical modeling cultures proposed by Leo Breiman, namely, data modeling and algorithmic modeling. Recently, we have also witnessed the great success of **deep learning** in protein structure prediction. In this review, we present a survey of the efforts for protein structure prediction. We compare the research paradigms adopted by researchers from different fields, with an emphasis on the shift of research paradigms in the era of deep learning. In short, the algorithmic modeling techniques, especially deep neural networks, have considerably improved the accuracy of protein structure prediction; however, theories interpreting the neural networks and knowledge on protein folding are still highly desired.

## What is protein structure prediction?

**Figure 1:**
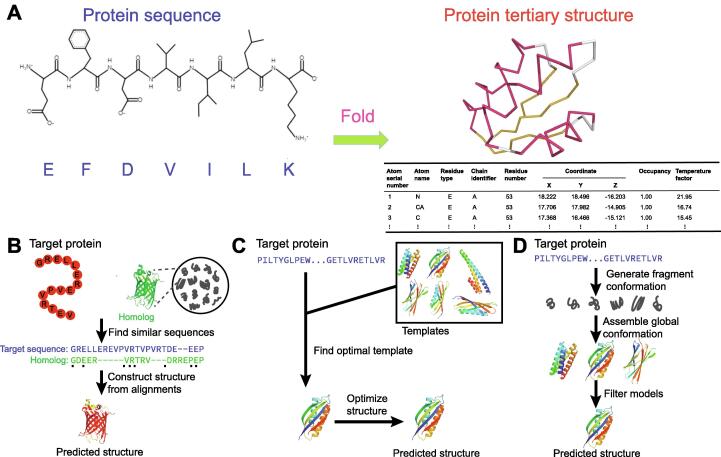
**Protein sequence, protein structure, and protein structure prediction** **A.** An example of protein sequence and its tertiary structure. Here, we show a C-terminal fragment of the ribosomal protein L7/L12 from *Escherichia coli* (PDB: 1CTF), which consists of a total of 74 residues linked via peptide bonds. The tertiary structure specifies the unique 3D coordinates of each atom in the relative position of the whole protein. Cartoon backbone representation is widely used to visualize protein tertiary structure. **B.** Homology modeling method for protein structure prediction. **C.** Threading method for protein structure prediction. **D.***Ab initio* prediction approach. PDB, Protein Data Bank; 3D, 3-dimensional.

Proteins are large biomolecules that perform essential functions within organisms, including transporting molecules, responding to stimuli, providing structure to cells, and catalyzing metabolic reactions [1]. A protein comprises one or more long chains of amino acid residues linked via peptide bonds. In the natural environment, a protein usually spontaneously folds into a specific tertiary structure (called native structure), in which each atom occupies a unique position in the 3-dimensional (3D) space of the molecule (Figure 1A). The main factors driving a protein to fold into its native structure are numerous non-covalent inter-residue interactions, including hydrophobic effects, hydrogen bonds, van der Waals forces, and ionic bonds [1], [2].

Protein structures are characterized by the regular conformation shape in some local regions. These regular, local structures, called protein secondary structures, are formed by the hydrogen bonds among amide groups of residues. The most prevalent secondary structure is the right-handed spiral *α-*helix, in which a backbone amino group donates a hydrogen bond with another backbone carbonyl group and the sequence distance between these two groups is 3.6 amino acids on average. Another common secondary structure is the β strand, which exhibits an almost fully extended conformation. Two or more β parallel or antiparallel strands connected by hydrogen bonds among them form a β*-*sheet. Figure 1A shows a C-terminal fragment of the ribosomal protein L7/L12 from *Escherichia coli* in Protein Data Bank (PDB: 1CTF) as an example, which consists of three *α-*helices and three β strands. The accurate prediction of the secondary structure of a protein provides important information of its tertiary structure [3], [4].

Cognizance of the native structures of proteins is highly desirable, as protein functions are determined mainly by their tertiary structures. The native structures of proteins can be experimentally determined using nuclear magnetic resonance, X-ray crystallography, and cryogenic electron microscopy [5]. However, these experimental technologies are usually expensive and time-consuming and thus cannot keep pace with the rapid accumulation of protein sequences. In contrast to these structure determination technologies, the protein structure prediction approaches, *i.e.*, predicting protein structure purely from protein sequences using computational techniques, are highly efficient [6], [7]. It should be pointed out that the prediction of structure for a protein purely from its sequence is feasible as the structure information is essentially embedded in protein sequence according to the Anfinsen’s dogma, *i.e.*, an unfolded protein usually refolds to its native structure when restoring the protein to an aqueous environment under appropriate conditions [8].

## Protein structure prediction approaches: rationale, categories, and representatives

Accurate prediction of protein structures relies heavily on a deep understanding of the protein folding process and the relationship between protein sequences and native structures. The native structure of a protein is the state in which the protein takes the lowest free energy and nearly all residues fit perfectly with their local structural environments [9].

The evolutionary history of a query protein, which is usually described using the multiple sequence alignments (MSAs) of its homologies, provides abundant information to infer its native structure. Specifically, the residues with critical roles in stabilizing structure are relatively conserved; in contrast, the residues in contact tend to co-mutate during the evolutionary process [10].

Protein sequence and structure can be represented in various ways. We can represent the sequences of homology proteins as MSAs or position sequence scoring matrix (PSSM). We can further process MSAs into profile hidden Markov models or even conditional random fields to emphasize the correlations among residues. Similarly, a protein structure can be depicted using the coordinates of all its atoms, the torsion angles associated with each Cα atom, or the distances between residue pairs.

Most of the existing approaches accomplish structure prediction by effectively exploiting the sequence–structure relationship and the evolutionary information carried by the homologous proteins of the target protein. The current approaches can be divided into template-based modeling (TBM), which requires template proteins, *i.e.*, the proteins with solved structures, and free modeling (FM, also known as *ab initio* approaches), which do not rely on any templates. The TBM approaches can be further divided into homology modeling and threading. The basic idea and representative software implementations of these approaches are described in detail as follows.

### Homology modeling methods

The rationale underlying the homology modeling approaches is that protein structures are more conserved than sequences during the evolutionary process, and homology proteins, especially the close homology proteins, usually share similar structures; therefore, we can construct a structure for a target protein by referring to the structures of its homologies (Figure 1B). The widely used strategy to identify homologies of a target protein is “sequence–sequence“ alignment. Two proteins will be considered homology proteins if their sequence alignment shows sufficiently high sequence similarity [11].

Based on the acquired alignment of the target protein with a homology template, its structure can be constructed by running structure modeling tools, such as MODELLER [12]. In this strategy, both target proteins and templates are represented using their sequences alone [13] or sequences of homology proteins. The sequences of homology proteins, which are organized as MSAs, PSSM, and profile hidden Markov model [13], [14], [15], have proven to be effective to increase the sensitivity of homology identification.

The representative homology identification or homology modeling software tools include PSI-BLAST [13], PDB-BLAST [16], SAM-T99 [17], FFAS [18], ORFeus [19], HMMER [14], and HHpred [15]. Generally speaking, the homology identification/modeling approaches work perfectly when a homology template can be identified with notable similarity to the target protein (sequence identity exceeding 30%).

### Threading methods

Unlike the homology modeling technique, which seeks to find a template with significantly high similarity to the target protein, the threading approaches aim to find a protein with the same structural fold (Figure 1C). Thus, the core step of threading approaches is to calculate the compatibility of the target protein sequence with the structures of templates, which relies on “sequence–structure” alignment rather than “sequence–sequence” alignment as performed by the homology modeling approaches [20], [21].

The representative threading tools include PROSPECT [22], RAPTOR [20], SAM-T02 [23], mGenTHREADER, 3D-PSSM, SPARKS [24], TASSER [25], CNFpred [26], MRFAlign [27], DeepThreader [21], and ProALIGN [28]. Compared with homology modeling approaches, threading approaches usually yield more accurate prediction results as they exploit the structural information of templates. Another advantage of threading approaches is that they apply when only remote homology templates are available for the target protein.

### *Ab initio* prediction methods

Most *ab initio* prediction approaches are based on the first principle, which states that, in the natural environment, a protein tends to adopt the structural conformation with the lowest free energy. Thus, structure prediction can be accomplished through minimizing an energy function or directly simulating the folding process (Figure 1D) [29], [30], [31].

Direct simulation of the protein folding process is attractive and promising: the Shaw group simulated the folding process of 12 representative proteins using a single physics-based energy function [32]. The simulation results of these proteins enabled them to reveal several principles governing the folding process. However, this strategy is precluded by the vast computation cost, partly due to the inaccurate energy function and the enormous space of possible structural conformations. For example, the Shaw group performed the molecular dynamics simulation on a specially-designed high-performance computer [33], and Duan et al. acquired the simulation of one millisecond for a short protein with only 36 amino acids, which costed two months on a Cray machine with 256 processors [34].

It is a challenging task to design accurate energy functions for *ab initio* approaches. Most of the widely used energy functions are hand-crafted and thus require considerable expertise from designers. For example, Rosetta uses a full-atom energy function that consists of over 140 energy terms to depict various aspects of protein structures [35], and most of the Rosetta energy terms are knowledge-based. Besides the hand-crafted energy functions, an alternative way is to apply machine learning techniques to design energy terms [36], [37] or find the optimal weights of energy terms [38]. For example, trRosetta uses an energy function derived from the inter-residue distances predicted by a deep neural network [36]. The energy functions thus constructed usually have only a few effective energy terms and can avoid the inaccuracy of hand-crafted energy functions.

The representative *ab initio* prediction software tools include FRAGFOLD [39], FALCON [40], Rosetta [29], RoseTTAFold [41], I-TASSER [42], QUARK [43], trRosetta [36], AlphaFold [37], AlphaFold2 [44], ProFOLD [45], RGN2 [46], ESMFold [47], and OmegaFold [48].

### Representative approaches to protein structure prediction

**Figure 2:**
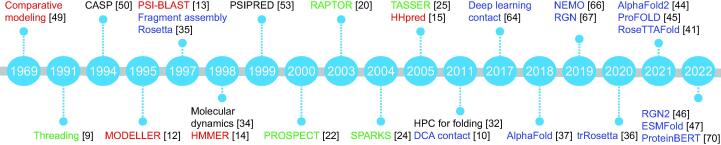
**Chronological diagram of the representative approaches to protein structure prediction** Here, homology modeling approaches are shown in red, template-based approaches are shown in green, *ab initio* approaches are shown in blue, and other techniques are shown in black.

We summarize in Figure 2 the representative approaches to protein structure prediction and describe them briefly as follows.

In 1969, Browne et al. proposed the comparative modeling strategy for structure prediction, which identifies homology templates of a target protein through comparing it with protein sequences, and then constructs structure for the target protein by referring to the structures of its homology templates [49]. This study inspired researchers to pay more attention to developing algorithms for homology identification.

In 1991, Bowie et al. studied the refolding problem (*i.e.*, designing a protein sequence that can fold into a desired structure) and proposed the threading strategy for structure prediction. The key concept of this strategy is “sequence–structure” alignment, which is calculated by evaluating the fitness of each residue in the target protein with the local structural environment of its aligned residue in templates [9]. The concept of “local structural environment” has been widely used in subsequent studies on threading approaches and protein design.

In 1994, Moult et al. initiated the biennial Critical Assessment of Structure Prediction (CASP) competition. The CASP competition provides research groups with an opportunity to objectively assess their prediction approaches and has greatly advanced the development of the field [50].

In 1995, Šali et al. developed MODELLER, a tool to construct a structure model that satisfies spatial restraints of residues as much as possible [12]. The spatial restraints can be derived from the alignment of the target protein with a template with known structure; and thus, MODELLER is widely used to build models together with homology identification or threading approaches.

In 1997, Altschul et al. developed PSI-BLAST [13], the successor of BLAST [51], for sequence comparison and homology identification. PSI-BLAST uses a position-specific scoring matrix to calculate the similarity between two protein sequences and is much more sensitive to weak but biologically relevant sequence similarity than BLAST.

In 1997, Baker et al. proposed the fragment assembly strategy and implemented an *ab initio* prediction software toolRosetta [35], [52]. Rosetta constructs the structure for a target protein using the combination of structure fragments (9-mers or 3-mers) extracted from the known structures deposited in the PDB. Rosetta uses the Monte Carlo search technique and fragment replacement strategy to find the combination of structure fragments with the lowest free energy. An advantage of the fragment assembly strategy is that structure fragments implicitly characterize subtleties of local structure preferences and thus significantly resolve the difficulties of designing energy functions.

In 1999, Jones developed PSIPRED [53], a two-stage neural network to predict the secondary structure of a target protein based on its sequence profile. PSIPRED slides a 15-residue window along the target protein sequence, feeds into the neural network the profile of the residues within this window, and uses this information to predict the secondary structure type of the central residue of the window. PSIPRED shows better prediction accuracy than statistical approaches, say Chou-Fasman [54] and GOR [55]. Recent advances in protein secondary structure include DNSS [56], DeepCNF [57], SPIDER3 [58], and SPOT-1D [59].

In 2004, Zhou et al. developed SPARKS, an approach that combines a residue-level potential, sequence profile, and secondary structure information for fold recognition [24]. SPARKS achieves better accuracy and sensitivity than PSI-BLAST in sequence–sequence alignment and detecting similarities at family, superfamily, and fold levels.

In 2005, Zhang et al. designed an *ab initio* prediction tool called I-TASSER [6], [42]. Unlike Rosetta, which utilizes fixed-length structure fragments, I-TASSER uses variable-length fragments derived from threading results and exploits the estimated inter-residue distances. Xu et al. further developed another *ab initio* prediction software called QUARK [60].

In 2005, Söding et al. developed HHpred, a tool for remote protein homology detection and structure prediction [15]. HHpred is featured by representing both target protein and template as hidden Markov models and then comparing the two hidden Markov models to yield protein alignment.

In 2008, Vassura et al. reported that the backbone structure of a protein can be accurately reconstructed from the inter-residue contacts within this protein through iteratively tuning the coordinates of atoms [61]. Here, two residues are viewed as “in contact” if the distance between their Cα atoms is less than a threshold (ranging from 3 Å to 12 Å in the study). This study has opened a new possibility to predict protein structure using inter-residue contacts or inter-residue distances.

In 2011, Marcos et al. proposed the direct coupling analysis (DCA) technique to infer co-evolution events from MSAs of target protein, in which protein sequences can be modeled using Markov random fields or Gaussian distribution [10]. The DCA technique has the advantage of removing the potential transitivity among co-mutations. Using the DCA technique, CCMpred [62] and PSICOV [63] have shown excellent performance in predicting inter-residue contacts. These successes have inspired the community to further predict inter-residue distances and then apply the predicted inter-residue contacts and distances in protein structure prediction.

In 2017, Wang et al. proposed to apply the deep learning technique to refine the predicted residue contacts by CCMPred. Using a 60-layer ResNet to consider all residue pairs simultaneously, the proposed approach RaptorX-Contact shows excellent accuracy in predicting inter-residue contacts [64].

In 2018, Senior et al. proposed AlphaFold [37], achieving protein prediction with better accuracy and robustness by generating the final structure using optimization rather than sampling. The optimization is based on a potential of mean force induced from the predicted distance map of the target protein using a simple gradient descent algorithm.

In 2019, Mirabello et al. proposed rawMSA, an end-to-end model using raw MSAs as input. rawMSA borrowed the embedding idea from natural language processing, which maps a protein sequence into an adaptively learned continuous space. rawMSA showed promising results in predicting solvent accessibility of residues and inter-residue contacts [65].

In 2019, Ingraham et al. proposed an end-to-end prediction approach called NEMO [66], which composes a neural energy function and an unrolled Monte Carlo simulator that simulates the folding process. NEMO showed the potential to make multimodal predictions and promising generalization ability.

In 2019, AlQuraishi proposed RGN, another end-to-end differentiable model via a neural network that optimizes both local and global geometry concurrently [67]. RGN does not exploit the co-evolution information; instead, it iteratively extends inter-mediate structure through appending a residue at each iteration step according to the predicted torsion angles.

In 2020, Mao et al. proposed GDFold, an approach to rapid protein structure prediction [68]. The approach predicts inter-residue contacts using a neural network with its architecture optimized through automatic search, and uses all of the predicted inter-residue contacts rather than considering the top-scored contacts only. The recent advance from the same lab also reveals that, by using conditional neural network, we can achieve a prediction accuracy comparable with the approaches using Transformer.

In 2020, Yang et al. proposed the trRosetta algorithm, which uses deep neural networks to estimate inter-residue distance, dihedral torsion angles, and relative orientation of long-distance residue pairs. trRosetta constructs an energy function using the predicted inter-residue distances and orientations and then searches for the structure with the lowest energy. Experimental results suggest that although the energy function constructed in this manner contains only a few energy terms, it outperforms the hand-crafted energy functions [36].

In 2021, Ju et al. proposed CopulaNet, a neural network model that directly learns inter-residue distances from MSAs rather than refines the predicted distances generated by statistics techniques [45]. ProFOLD, a software tool implementing CopulaNet, shows superiority over other approaches on CASP13 target proteins, thus demonstrating the power of directly learning inter-residue distances from MSAs [45].

In 2021, Rao et al. proposed a Transformer framework that learns protein structure and function from sets of homologous sequences organized as MSA. The model, called MSA Transformer, interleaves row- and column-attention to exploit the conservation of residues and equivalence of aligned residues across the input sequences. This model showed outstanding performance in predicting inter-residue contacts and protein structure [69].

In 2021, DeepMind announced AlphaFold2 [44], an end-to-end model for protein structure prediction that can predict protein structures with atomic accuracy regularly. AlphaFold2 consists of a module to encode MSAs and build pairwise representations, a 3D rotation equivariant network to build structure, and a recycling mechanism to iteratively improve structure prediction. AlphaFold2 also reports a measure called predicted Local Distance Difference Test (pLDDT) as confidence of the predicted model. AlphaFold2 achieves great success as it can predict the structure for many proteins with an accuracy comparable to experimental determination technologies [44].

In 2021, Baek et al. proposed RoseTTAFold [41], which predicts structure using a three-track network that integrates information from the sequence, distance map, and 3D coordinates. Unlike AlphaFold2 using an invariant point attention module, RoseTTAFold uses an SE(3)-Transformer to reconstruct protein 3D structure from the inter-residue distances. RoseTTAFold shows an accuracy close to AlphaFold2 although it uses much less computation power for training neural network.

In 2022, Brandes et al. proposed ProteinBERT, a language model to learn the conditional probability of observing a residue given its neighbors from many protein sequences [70]. ProteinBERT has shown excellent performance in various tasks, including secondary structure prediction, remote homology identification, and stability prediction.

In 2022, Chowdhury et al. proposed RGN2, a method for single-sequence structure prediction, *i.e.*, predicting structure for a target protein without using any of its homology proteins [46]. RGN2 uses a protein language model, AminoBERT, to learn latent structural information from 260 million proteins. Yu et al. developed ProFOLD Single, an improved version of ProFOLD [45], for single-sequence protein structure prediction. Wu et al. reported OmegaFold, another method for this objective [48]. These approaches are particularly suitable for orphan and rapidly evolving proteins, for which homology proteins are insufficient to build high-quality MSAs.

In 2022, Kandthil et al. proposed DMPfold2, an end-to-end prediction approach that reduces the preprocessing of target MSA and directly yields main chain coordinates. The core idea of DMPfold2 includes per-column and alignment embedding to infer inter-residue distances, as well as a learnable multidimension scaling (MDS) module to build main chain coordinates from the distances [71].

In 2022, Lin et al. proposed an extremely large protein language model ESM-2 with 15 billion parameters and then developed the prediction software ESMFold [47], even larger than the language models used by OmegaFold (670 million parameters). These studies demonstrated the power of the language model in protein structure prediction.

**Figure 3:**
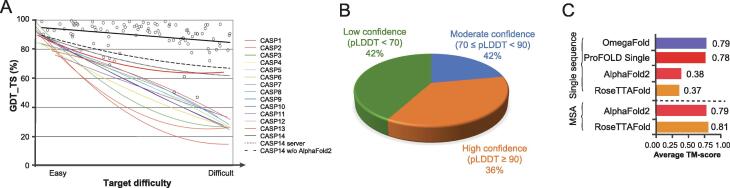
**Performance of representative approaches to protein structure prediction** **A.** Performance of the prediction approaches in previous CASPs. Trendlines indicate the agreement of the target protein backbone for the best-predicted structures with that of the native structures in the last 14 CASP rounds; open circles indicate the individual data points for CASP14. Target difficulty is based on sequence and structural similarity to existing experimental protein structures, which was adapted from [50] with permission. **B.** Prediction performance of AlphaFold2 for 20,296 human proteins covering 10,537,122 residues. For each protein, AlphaFold2 outputs a pLDDT score as an estimation of the prediction quality. For nearly 36% of proteins, AlphaFold2 predicts their structures with high confidence (pLDDT ≥ 90). The data were taken from [72]. **C.** The performance of the prediction approaches using MSAs or a single sequence as input. On 29 selected CASP-free modeling targets, AlphaFold2 and RoseTTAFold show excellent accuracy when using MSAs of query proteins as input. However, their performances decrease sharply when using a single sequence of query protein as their only input. In contrast, OmegaFold and ProFOLD Single, the approaches specially designed for single-sequence prediction, achieve high accuracies that approximate the approaches using MSAs. It should be noted that the accuracy of ProFOLD Single is acquired from CASP14 target proteins to avoid overlapping between training and test data, which was adapted from [48] with modifications. CASP, Critical Assessment of Structure Prediction; GDT_TS, Global Distance Test-Total Score; MSA, multiple sequence alignment; pLDDT, predicted Local Distance Difference Test.

The organizers of the CASP competitions analyzed the prediction approaches that attended CASP competitions and attributed the advance in prediction accuracy to the following key technologies: (1) the fragment assembly and replacement strategy for structure prediction; (2) predicting inter-residue contacts based on co-evolution information; (3) the application of deep learning technique, say ResNet, to predict inter-residue distances; and (4) predicting inter-residue distance using Transformer, and end-to-end prediction of protein structures (Figure 3A) [49].

Protein structure prediction has attracted scientists from multiple fields, including biochemistry, medicine, physics, mathematics, and computer science, and these scientists employed different research paradigms to attack the same problem. Here, we compare these research paradigms to understand their strengths and limitations.

## Physicists’ research paradigm

Physicists generally are highly interested in understanding the principles governing the protein folding process. In 1969, Levinthal posed a paradox: a protein might have an astronomical number of possible structural conformations due to its massive number of degrees of freedom of the structure. Suppose the protein reaches its native structure by examining all possible conformations (also known as “unbiased random search”). In this case, it will require a time longer than the universe’s age, which conflicts with the quick folding process occurring within seconds or even milliseconds. Levinthal himself explained the quick folding process as the result of “local interactions”, *i.e.*, local amino acids form stable interactions and serve as nucleation points, significantly limiting the search space and thus guiding the further folding process [73]. Levinthal also stated that there are well-defined folding pathways to the native state [30].

To investigate how the Levinthal paradox can be resolved, Šali et al. conducted a Monte Carlo simulation for a 27-bead self-avoiding chain on a cubic lattice. The simulation suggested a three-stage model of protein folding: folding starts with a rapid collapse from the random unfolded state to a random semi-compact globule, proceeds by a slow random search to find a transition state, and finally folds rapidly to the native state. The semi-compact globules and transition states differ in the number of native contacts. This model resolves the Leventhal paradox as it contains the following key elements: (1) the reduced number of conformations needed to be searched in the semi-compact globule, and (2) the existence of multiple transition states [74].

In the 1990s, a new view of protein folding replaces the concept of “folding pathways” with “folding funnels” in the energy landscape. Intuitively, folding is treated as water trickling down the mountainside of complex shapes rather than flowing along a single tunnel. This new view emphasizes an ensemble of proteins that fold parallelly rather than a single structure and a specific folding pathway [31], [75], [76].

**Figure 4:**
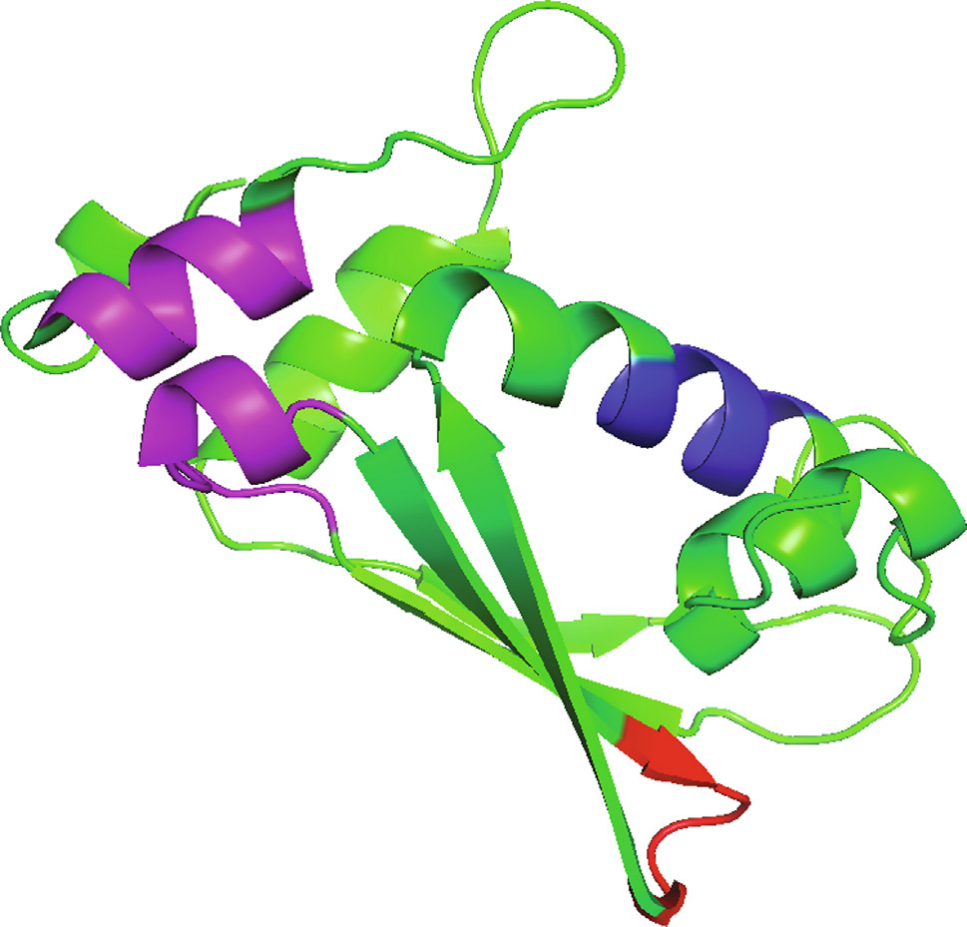
**Strong structural signals in protein Se0862 (PDB: 6UF2)** Three types of regions that might carry strong structural signals, including single helical turn (blue), β-turn (red), and a pair of secondary structural elements with contact between them (purple).

In 2015, Zheng [77] investigated protein folding process by searching strong structural signals in protein sequences, which might be a single helical turn, β-turn, or two secondary structure elements in contact (Figure 4). In the case of *α*-helices, folding starts by rapidly forming a single helical turn containing three or four residues with strong structural preference, which guides the neighboring residues to fold into the entire helix. Following this idea, Wang et al. identified dozens of residue pairs with significantly strong structural preferences, which can be categorized as hydrophobic residue pairs, residues with opposite charges, and proline-containing residue pairs (Table 1). The identified residue pairs perfectly agree with the observations from mutated proteins and molecular dynamics simulation experiments. These residue pairs have also shown excellent performance in secondary structure prediction for a single sequence without referring to any homology protein [78].

**Table 1:** **The key residue pairs in helices with significantly high odds ratio extracted from 3206 protein domains**

**A_i_ –A_i+3_**			**A_i_ – A_i+4_**		
**Residue pair**	**Count**	**Odds ratio**	**Residue pair**	**Count**	**Odds ratio**
E–K	1980	1.67	K–D	843	2.25
I–E	1336	1.59	Q–D	640	2.24
E–R	1760	1.57	K–E	1558	1.94
P–V	508	1.50	R–E	1392	1.79
D–R	901	1.49	E–K	1889	1.69
N–K	516	1.42	I–I	940	1.53
D–K	904	1.41	E–R	1549	1.52
R–E	1083	1.33	F–V	570	1.48
K–D	504	1.26	P–E	564	1.43
L–Y	834	1.20	A–A	3699	1.43
V–A	2013	1.17	G–A	1276	1.40
L–M	633	1.17	R–D	508	1.39
K–Q	560	1.17	P–A	856	1.38
E–E	1565	1.17	V–I	981	1.36
L–L	3124	1.16	Q–E	826	1.36

*Note*: A_i_–A_i+3_ and A_i_–A_i+4_ represent a pair of residues residing in the same helix with 3 and 4 residues apart, respectively. These residue pairs are highly likely hydrogen bonding as helix turns with 3*.*6 residues on average. For A_i_–A_i+3_, odds ratio of a residue pair *x*–*y* is defined as $\frac{p_{\mathrm{xy}}}{p_{x\cdot }p_{\cdot y}}$, where $p_{\mathrm{xy}}$, *p_x_.*, and *p._y_* denote in all residue pairs in the same helix with 3 residues apart, the probability that the first residue is *x* and the second residue is *y*, the probability that the first residue is *x*, and the probability that the second residue is *y*, respectively. For A_i_–A_i+4_, odds ratio is defined likewise, considering residues with 4 residues apart instead of 3. The 3206 protein domains are from the SCOP70 database. SCOP, Structural Classification of Proteins.

Previous studies emphasized the fitness of a residue with its local structural environment in the native structure [9]. Complementing these studies, Zheng reported the existence of residues unfitting with their local structural environments. These residues, although unfavorable from the local point of view, are usually beneficial to stabilize the global structure. Zheng also emphasized the formation of coarse topology in protein folding, which can be exploited to improve protein structure prediction [77].

## Statisticians’ research paradigm

Statistics starts with observed data and think of the data as being generated by a model that takes a vector of variables as input and yields a collection of response variables. Statisticians usually start by assuming a statistical model of the observed data when faced with a practical problem. This research paradigm, denoted as “data modeling” or “the first statistical culture” by Leo Breiman [79], was almost exclusively used by the statistics community.

**Figure 5:**
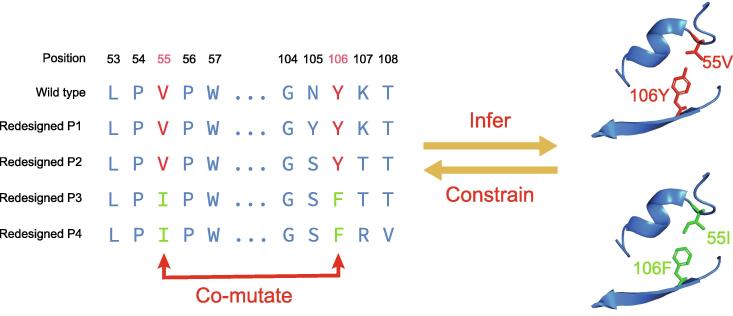
**An example ofaninter-residue contactin GFP (PDB: 4EUL) and co-mutations observed in its homologs** Two residues in contact 55V–106Y (shown in red) co-mutate to 55I–106F (in green) to maintain the contact between them; and thus, in turn, the co-mutations observed in homologous proteins can be exploited to infer inter-residue contacts. To demonstrate this, we use ProDESIGN-LE2, a protein sequence design method, to design four sequences (P1–P4) for the structure of GFP. As the design process of ProDESIGN-LE2 resembles the evolution of the target protein, the resulting designed sequences could be used as an approximation of the homologies of target proteins. ProDESIGNE-LE2 is an improved version of ProDESIGN-LE [79]. GFP, green fluorescent protein.

The data model has been successfully applied in protein structure prediction, especially the prediction of inter-residue contacts and distances. Knowing inter-residue distances is a critical step as protein 3D structures can be accurately reconstructed from residue distances. Currently, nearly all effective methods to predict inter-residue distances exploit the co-mutation of residue pairs occurring during evolution. Briefly speaking, for an ancestor protein with two residues in contact, the two residues in its descendant proteins at the corresponding positions always show a strong tendency to co-mutate to stabilize the entire structure. These co-mutations, also known as co-evolutionary events, can be exploited to predict inter-residue contacts (Figure 5) [80].

To reduce the false-positive prediction derived from the transitivity among co-mutations, Marcos et al. proposed the DCA technique, which models the entire protein sequence rather than considering residue pairs individually [10]. The implementations of the DCA technique differ in their assumptions of the distribution of protein sequences. For example, CCMPred [62] assumes that each homology protein sequence (denoted as *x*_1_, *x*_2_, ···, *x_n_*) of the target protein is generated from a Markov random field shown below:

(1) $$P\left(x_{1},x_{2},\cdots ,x_{n}\right)\propto \text{exp}\left(\sum_{i=1}^{n}s_{i}\left(x_{i}\right)+\sum_{i<j}h_{\mathrm{ij}}\left(x_{i},x_{j}\right)\right)$$

Here, the singleton term *s_i_*(*x_i_*) represents the local preference of the *i*-th residue, and the doubleton term *h_ij_*(*x_i_*,*x_j_*) represents the coupling strength between the *i*-th and *j*-th residues. These parameters can be determined using the maximum likelihood technique, and the residue pairs with high coupling strength will be reported as residues in contact.

Unlike CCMpred, PSISOV [63] assumes that the homology protein sequences (denoted as *n*-length vector *X*) are generated from a high-dimensional Gaussian distribution as follows:

(2) $$f\left(X\right)=\frac{1}{\sqrt{{\left(2\pi \right)}^{n}\left|\Sigma \right|}}\exp\left(-\frac{1}{2}{\left(X-\mu \right)}^{T}\Sigma^{-1}\left(X-\mu \right)\right)$$

Here, *µ* denotes the expectation of *X*, and Σ denotes the covariance matrix. The inverse of Σ, called precision matrix, contains direct coupling information between residues and thus is used to infer inter-residue contacts.

Although effective for some proteins, the data modeling paradigm usually suffers from the misassumption of data generating process and statistical data distribution. Ju et al. provide a concrete example: when assuming Gaussian distribution for the two proteins, we will acquire the same co-variance matrix and thereafter, the same prediction of inter-residue contacts, despite the marked difference between their structures and sequences [45].

## The shift of research paradigms in the era of deep learning

In the era of deep learning, a new research paradigm has emerged, which applies deep neural networks to learn the rules of protein sequences [47], predict protein secondary structures [56], [57], [81], infer inter-residue contacts or distances [36], [45], [64], [82], assess protein model quality [83], and even construct protein structures from sequences in an end-to-end manner [44]. This research paradigm, denoted as “algorithmic modeling” or “the second statistical culture” by Leo Breiman, differs from the data modeling paradigm in the fact that it uses the empirical distribution embedded in large-scale data rather than an assumed distribution [79], [84]. Thus, the algorithmic modeling paradigm gains an advantage in avoiding the misassumption of data generating process and distribution of the observed data.

The early applications of deep neural networks in protein structure prediction include: (1) CMAPpro, an approach aiming to improve contact prediction using deep learning [82]; (2) DL-Pro, a purely geometry-based deep learning algorithm to assess the quality of a protein model according to its inter-residue distance matrix [83]; (3) DNSS, a method to predict protein secondary structure using deep learning with sequence profile as input [56]; (4) SPIDER, a deep neural network that predicts backbone’s local geometric features, say *θ* and *τ* angles [85]; and (5) RaptorX-Contact, an approach to the prediction of inter-residue contacts [64]. Unlike PSICOV deriving inter-residue contacts from the precision matrix, RaptoX-Contact uses a ResNet (with up to 60 layers) to learn the principles of inter-residue contact map and refine it to approximate the ground truth. Experimental results demonstrate the advantages of RaptorX-Contact over CCMpred and PSICOV.

The protein language model is another representative of the algorithmic modeling paradigm: instead of assuming a high-dimensional distribution of protein sequences, the protein language model uses deep neural networks, say Transformer, to learn the latent rules of protein sequences, including the appearance of a specific residue type conditioned on its prefix or its two neighbors [47], [70]. The language models trained on a large-scale protein sequence dataset, say ProteinBERT and ESM, have shown excellent performance in predicting protein structure and functions.

Sequence–structure alignment can also be learned from the inter-residue distance map: Kong et al. applied deep convolutional networks to recognize the frequently occurring patterns (called alignment motifs) from inter-residue distance maps and then used the identified motifs to construct sequence–structure alignments for threading. The software implementation of this idea, ProALIGN, has shown superiority over other threading tools using a hand-crafted scoring function [28].

Most of the above-mentioned approaches use human-engineered pipelines comprising multiple complex components. The prediction of tertiary structure from sequence using a single step is attractive. Recent advances, including RGN [67], NEMO [66], and AlphaFold2 [44], apply deep neural networks to predict structures in an end-to-end fashion. The advantage of the end-to-end fashion lies in the back-propagation from structure directly to sequence, which also means that it can learn the sequence–structure relationship directly from data rather than relying on an artificial assumption.

Continuous efforts have been devoted to reorienting or extending AlphaFold2 and have achieved some prominent successes. For example, Tsaban et al. showed that AlphaFold2 can be applied to model peptide–protein docking [86]. Using AlphaFold2, they predicted peptide–protein structures at high accuracy, identified interface hotspots, and modeled binding-induced conformational changes. Bryant et al. showed that AlphaFold2 can be applied to predict heterodimeric protein complexes structures with acceptable quality by optimizing input MSAs [87].

The successes in protein structure prediction could be helpful for structural biologists. Kryshtafovych et al. showed that among seven unsolvable targets, four of them get resolved with the aid of the prediction results of AlphaFold2 [88]. Slavin et al. used the domain structures predicted by AlphaFold2 together with cross-linking data acquired using mass spectrometry and finally obtained a single consistent all-atom model of the full-length Nsp2 protein from SARS-CoV-2 [89]. Mccoy et al. showed that the prediction of AlphaFold2 can be used to improve molecular replacement phasing in crystallography [90]. Fontana et al. attained a nearly complete structure of a megadalton protein complex, the cytoplasmic ring of the nuclear pore, by integrating medium-resolution density maps obtained using single-particle cryo-electron microscopy analysis and structure predictions from AlphaFold2 [91]. Terwilliger et al. developed an approach that synergistically combines the AlphaFold2 prediction procedure and measuring experimental density maps. This strategy achieved better prediction accuracy than the approaches that consider sequence alone or simply rebuild a model guided by experiment data [92].

These progresses have demonstrated the advantages of the algorithmic modeling paradigm in the era of deep learning and big data.

## Evaluation of the representative prediction approaches

**Figure 6:**
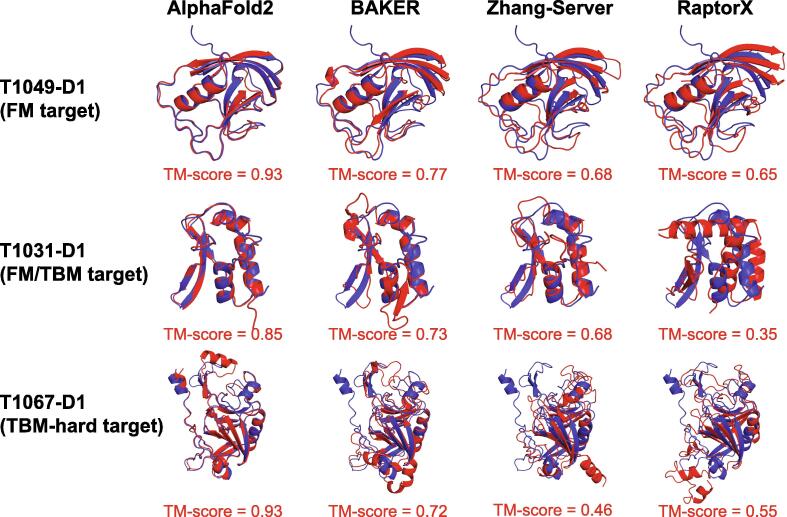
**Predicted structures for CASP14 targetsT1049-D1, T1031-D1, and T1067-D1 by AlphaFold2, BAKER, Zhang-Server, and RaptorX** For each representative target (in rows) in a target group defined in CASP14 and each predicting method (in columns), the alignment between the predicted structure (red) and the native structure (blue) is shown. Targets are mainly classified into TBM and FM categories using their prediction quality and template detectability. TBM, template-based modeling; FM, free modeling; TM-score, template modeling score.

We evaluated several representative prediction approaches on the CASP14 targets. As shown in Table 2, AlphaFold2 achieved an average TM-score of 0.91 on all the 87 CASP14 targets, higher than CASP human groups (BAKER: 0.74, FEIG-R2: 0.71) and servers (Zhang-Server: 0.70, BAKER-ROSETTASERVER: 0.64, Yang-Sever: 0.66). AlphaFold2 achieved a high performance of 0.84 on the 23 free modeling targets. Figure 6 shows the predictions for three CASP14 targets as an example.

**Table 2:** **Performance of representative methods to protein structure prediction on CASP14 targets**

**Method**	**All** **(*n*=87)**	**TBM-easy** **(*n*=22)**	**TBM-hard** **(*n*=28)**	**FM/TBM** **(*n*=14)**	**FM** **(*n*=23)**	**Web link**
AlphaFold2	**0.91**	**0.95**	**0.91**	**0.92**	**0.84**	
BAKER	0.74	0.84	0.75	0.74	0.61	
FEIG-R2	0.71	0.84	0.71	0.71	0.57	
Zhang-Server	0.70	0.85	0.70	0.69	0.56	https://zhanggroup.org/I-TASSER
BAKER-ROSETTASERVER	0.64	0.82	0.71	0.67	0.38	https://robetta.bakerlab.org
Yang-Server	0.66	0.83	0.69	0.68	0.44	https://yanglab.nankai.edu.cn/trRosetta

*Note*: Here, the table shows each method’s TM-scores in each group of targets. Target groups are defined in CASP14. Targets are mainly classified into TBM and FM categories using their prediction quality and template detectability. The highest TM-score in each group is shown in bold. The target count in each group is shown below the group name. Method names are names of participants in CASP14, including those in the human group (AlphaFold2, BAKER, and FEIG-R2), and those in the server group (Zhang-Server, BAKER-ROSETTASERVER, and Yang-Server). For methods in the server group, we also provide their corresponding server weblinks. CASP14, the 14th Critical Assessment of Structure Prediction; TBM, template-based modeling; FM, free modeling; TM-score, template modeling score.

Recently, AlphaFold2 released the predicted structures for 20,296 human proteins [85]. As shown in Figure 3B, around 36% of these predictions are highly confident with pLDDT ≥ 90, around 22% are moderately confident (70 ≤ pLDDT *<* 90), and around 42% are of low confidence (pLDDT < 70). Recent studies also showed that AlphaFold2 and RoseTTAFold work fine when sufficient homology proteins can be found: they achieved high accuracy (TM-score: 0.79 and 0.81) on 29 CASP13/14 free modeling targets. However, when target sequences are used as their only input (*i.e.*, no homology protein), the accuracy of these two approaches reduced sharply to 0.38 and 0.37, respectively [48]. In contrast, OmegaFold and ProFOLD Single, the approaches specially designed for structure prediction with single-sequence input, exhibited an accuracy of 0.79 and 0.64, respectively (Figure 3C). These results demonstrate the potential of the two approaches to approximate the prediction accuracy acquired using sufficient homology proteins as input.

## Conclusion and perspectives

Through the review, we have witnessed the shift of research paradigms from data modeling to algorithmic modeling in protein structure prediction. Recent progress has highlighted the power and special features of deep learning technique: the protein language models, say ProteinBERT and ESM, can effectively learn the rules underlying protein sequences; ResNet and convolutional networks can be used to precisely infer inter-residue contacts and distances; end-to-end networks can even learn the sequence–structure relationship and after that accurately predict structures for a large variety of proteins. From our perspective, the deep learning technique can also be used to solve the following problems, which might be the focus of future studies.

### Single-sequence-based structure prediction

Anfinsen’s experiments [8] have provided strong evidence and sufficient supporting argument to the sequence–structure relationship: the structure information is almost completely embedded in protein sequences. Thus, a protein structure can be accurately predicted from its sequence without referring to its homologies. The main challenges of this task lie in the understanding of the sequence–structure relationship and the construction of MSAs to approach the true evolutionary history of the target protein.

The leading approaches, including AlphaFold2 and RoseTTAFold, require high-quality MSAs comprising homology proteins and perform poorly when homology proteins are unavailable. The recent approaches, say OmegaFold and ProFOLD Single, have shown promising potential. In our opinion, the major reasons might be the application of language models to learn the rules of protein sequences and the efforts to reconstruct MSAs, which essentially describes the evolutionary history of target proteins. We are confident that, using improved neural network architecture, single-sequence prediction will achieve the accuracy of the prediction approaches exploiting homology proteins.

### Accurate and efficient protein sequence design

Protein sequence design aims to design a protein sequence that can fold into a desired backbone structure. Protein sequence design is now widely applied in rational protein engineering, and increasing the design accuracy and efficiency is desired.

Protein sequence design is exactly the reverse of protein folding; and thus, deep learning techniques, which have proven successful in protein structure prediction, should also greatly facilitate protein sequence design. The main challenges include the understanding of the dependency of a residue type on its local structural environment, and the design of proteins with desired functionalities. Recent approaches, including ProteinMPNN [93], ProDESIGN-LE [79], and ABACUS-R [94], have exhibited promising results.

### Interpretation of the trained neural network

Despite the great achievements of deep learning techniques in protein structure prediction, we should be aware that increased precision in structure prediction does little to help improve our understanding of protein folding. This fact is essentially a mirror of “the gap between prediction and attribution” as pointed out by Efron [83]. The main challenges include the identification of the key changes of features in a trained neural network.

Efron has also proposed a possible solution to bridge prediction and attribution — using “traditional methods for analyzing a prediction algorithm’s output”. In the case of protein structure prediction, a feasible way is to interpret a trained neural network using a salience map [95] or causality and effect analysis. An alternative method is neural relationship inference, which has been successfully applied to analyze molecular dynamics trajectories [96]. These ways can identify the residues with critical roles in structure prediction or dynamics behaviors of proteins, thus improving our understanding of protein folding.

In summary, algorithmic modeling, *i.e.*, the second statistical culture, has become the dominant research paradigm for protein structure prediction and will definitely continue to play important roles in the future. However, to go beyond prediction toward attribution and to gain knowledge on protein folding, we need to integrate the first statistical culture with the second statistical culture.

## Competing interests

Fusong Ju and Jianwei Zhu are the current employees of Microsoft Corp. Qi Zhang is the current employee of Huawei Technologies Co., Ltd. All the other authors have declared no competing interests.

## CRediT authorship contribution statement

**Bin Huang:** Conceptualization, Resources, Writing – original draft, Writing – review & editing. **Lupeng Kong:** Conceptualization, Resources, Writing – original draft. **Chao Wang:** Conceptualization, Resources, Writing – original draft. **Fusong Ju:** Resources. **Qi Zhang:** Resources. **Jianwei Zhu:** Resources. **Tiansu Gong:** Resources. **Haicang Zhang:** Conceptualization, Resources. **Chungong Yu:** Conceptualization, Resources. **Wei-Mou Zheng:** Conceptualization, Resources, Writing – review & editing. **Dongbo Bu:** Conceptualization, Resources, Writing – original draft, Writing – review & editing, Supervision, Funding acquisition. All authors have read and approved the final manuscript.
